# MOR Is Not Enough: Identification of Novel mu-Opioid Receptor Interacting Proteins Using Traditional and Modified Membrane Yeast Two-Hybrid Screens

**DOI:** 10.1371/journal.pone.0067608

**Published:** 2013-06-28

**Authors:** Jessica Petko, Stephanie Justice-Bitner, Jay Jin, Victoria Wong, Saranya Kittanakom, Thomas N. Ferraro, Igor Stagljar, Robert Levenson

**Affiliations:** 1 Department of Pharmacology, Penn State College of Medicine, Hershey, Pennsylvania, United States of America; 2 Department of Biochemistry and Department of Molecular Genetics, University of Toronto, Toronto, Ontario, Canada; 3 Department of Psychiatry, University of Pennsylvania School of Medicine, Philadelphia, Pennsylvania, United States of America; National Institute on Drug Abuse, United States of America

## Abstract

The mu-opioid receptor (MOR) is the G-protein coupled receptor primarily responsible for mediating the analgesic and rewarding properties of opioid agonist drugs such as morphine, fentanyl, and heroin. We have utilized a combination of traditional and modified membrane yeast two-hybrid screening methods to identify a cohort of novel MOR interacting proteins (MORIPs). The interaction between the MOR and a subset of MORIPs was validated in pulldown, co-immunoprecipitation, and co-localization studies using HEK293 cells stably expressing the MOR as well as rodent brain. Additionally, a subset of MORIPs was found capable of interaction with the delta and kappa opioid receptors, suggesting that they may represent general opioid receptor interacting proteins (ORIPS). Expression of several MORIPs was altered in specific mouse brain regions after chronic treatment with morphine, suggesting that these proteins may play a role in response to opioid agonist drugs. Based on the known function of these newly identified MORIPs, the interactions forming the MOR signalplex are hypothesized to be important for MOR signaling and intracellular trafficking. Understanding the molecular complexity of MOR/MORIP interactions provides a conceptual framework for defining the cellular mechanisms of MOR signaling in brain and may be critical for determining the physiological basis of opioid tolerance and addiction.

## Introduction

Opioid agonist drugs are clinically important because they are potent analgesics. However, chronic exposure to opioid drugs causes profound changes in the brain, which may lead to opioid dependence. The analgesic and addictive properties of most clinically relevant opioid agonist drugs are mediated primarily via activation of mu-opioid receptors (MORs). The central role of MOR in mediating the effects of opioid agonist drugs was established using MOR knockout (KO) mice. MOR KO mice display significantly reduced sensitivity to both the analgesic and rewarding effects of opioids [1].

Regulation of MORs, like most G-protein-coupled receptors (GPCRs), occurs via multiple mechanisms including receptor desensitization, internalization, degradation, and recycling [2]. A number of studies have shown that MOR desensitization and receptor trafficking can increase the rewarding properties of opioid drugs, while reducing the development of opioid tolerance and addiction-like behaviors [3], [4], [5], [6], [7], [8], [9], [10], [11]. However, the specific molecular mechanisms that regulate these processes are largely unknown. Elucidating the mechanisms that regulate MOR signaling and trafficking is critical for determining the cellular response to opioid agonist drugs and for opening new avenues of investigation into the pharmacotherapy of pain management.

A fundamental principle that has emerged from decades of cell signaling research is that signaling molecules, including GPCRs, are assembled into macromolecular entities termed signaling complexes or signalplexes [12], [13]. It is now well established that receptor-protein interactions govern the structural and functional organization of GPCR-containing signalplexes [14], [15], [16], [17]. To date, more than 20 proteins that interact directly with the MOR (MORIPs; mu-opioid receptor interacting proteins) have been identified. These interacting proteins have been shown to play a role in regulation of MOR trafficking, subcellular localization, and signaling [18]. Additionally, activation of the MOR can affect the function of some of its interacting partners. For example, we have shown that morphine promotes the interaction between the MOR and WLS (Wntless/GPR177; a protein required for Wnt protein secretion), and this interaction serves to inhibit Wnt protein secretion from transfected mammalian cells [19], [20].

To better understand the potential role of MORIPs in the MOR life cycle, we have initiated traditional and modified yeast two-hybrid (Y2H) studies designed to identify novel constituents of the MOR signalplex. Previous interaction screens for MORIPs have primarily utilized the third intracellular loop (IL3) or the C-terminal tail (C-tail) of the MOR as bait [18]. Here we have utilized the second intracellular loop as well as the entire MOR to screen human brain cDNA libraries in order to expand the growing list of MORIPs. Using these approaches, we have identified ten novel MOR binding partners, validated their interaction with the MOR, and examined the expression of three of these proteins in the brains of morphine-treated mice. In addition, we investigated whether two newly identified MORIPS, SIAH1 and SIAH2, are involved in ubiquitination or proteolysis of MOR. Further functional characterization of MORIPs will serve to heighten our understanding of the mechanisms regulating MOR-mediated signaling and may help elucidate the underlying molecular basis of cellular response to opioid agonist drugs.

## Materials and Methods

### Traditional and MYTH Two-Hybrid Screens

The traditional Y2H screening method involves the reconstitution of the GAL4 transcription factor through the interaction of a bait protein fused to the GAL4 DNA binding domain and a prey protein (from a fetal brain cDNA library) fused to the transcriptional activating domain of GAL4 [21]. This method is biased for cytosolic and nuclear proteins, as the protein complex must be imported into the nucleus to activate transcription. Therefore, only the cytosolic portion of integral membrane proteins is usually employed in this type of screen. Traditional Y2H screens in this study were performed as previously described [22], [23] using the second intracellular loop (IL2; amino acids 166–187) of the MOR as bait to screen a fetal human brain cDNA library. The MOR-IL2 was cloned into the yeast GAL4 DNA binding domain expression vector pAS2-1 (Clontech, Palo Alto, CA), while the human fetal brain cDNA library was provided in the GAL4 activation domain vector pACT2 (Clonetech). Bait and prey plasmids were successively transformed into yeast strain MaV103 [22]. Transformation of yeast with the fetal human brain library produced ∼2×10^6^ transformants/µg of DNA on quadruple dropout plates (-Leu/−Trp/−His/−Ura; Clonetech) containing 3-amino-1,2,4-triazol (3AT). Interactions were assayed for β-galactosidase (β-gal) activity via the nitrocellulose lift method [22]. cDNAs were extracted from yeast colonies, sequenced, and subjected to Basic Local Alignment Search Tool (BLAST) analysis to determine their identities.

To identify additional MOR interacting proteins (MORIPs), a modified split-ubiquitin membrane yeast two-hybrid (MYTH) screen was performed as previously described [24]. The MYTH system uses the split-ubiquitin method, in which the reconstitution of ubiquitin is mediated by a specific protein-protein interaction. Ubiquitin-specific proteases cleave at the C-terminus of ubiquitin, which releases a transcription factor that can translocate to the nucleus and activate transcription of a reporter gene [25]. The unique advantage of MYTH is that full-length integral membrane proteins can be used as bait and are amenable to protein-protein interaction analyses in their natural membrane environment [26], [27]. For this study, full-length human MOR cDNA in the bait vector pCCW-STE (Dualsystems Biotech AG, Switzerland) and a human fetal brain library in the prey vector pPR3-N (Dualsystems) were sequentially transformed into *S. cerevisiae* reporter strain THY.AP4. Transformation of yeast with the human brain library yielded 6×10^6^ transformants/µg DNA on quadruple drop out plates (−Trp/−Leu/−His/−Ade; Clonetech) containing 3AT. Fifty transformants were positive for β-gal activity. These colonies were picked and their cDNAs extracted, sequenced and subjected to BLAST analysis. From this screen we identified four novel MORIPs (Table 1) that were subjected to further biochemical analysis.

**Table 1:** Identification of novel MORIPs.

MORIP	Bait^1^	Accession Number	Residues^2^
AUP1	IL2	NM_181575	321–410
CSN5 (COP9S5)	IL2	NM_066837	25–318
Cx37	MOR	NM_002060	93–249
DOK4	IL2	NM_018110	65–289
DOK5	IL2	NM_018431	40–306
KCNF1 (Kv5.1)	MOR	NM_002236	215–489
RanBP9	IL2	NM_005493	88–366
SIAH1	IL2	NM_003031	1–233
SIAH2	IL2	NM_005067	122–324
VAPA (Vap33)	MOR	NM_194434	86–236
WLS (GPR177)	MOR	NM_024911	451–541
Zyxin	IL2	NM_001010972.1	203–483

1 MORIPs were identified using either the full-length mu-opioid receptor (MOR) or second intracellular loop of the MOR (IL2) as baits in MYTH or traditional Y2H screens, respectively.

2 Residues indicate the amino acid residues of the protein fragments recovered in the Y2H screens.

To map sites of interaction between the MOR and the newly identified MORIPs, each MOR intracellular loop (IL) was tested for interaction with individual MORIPs using the traditional Y2H method. MOR-IL domains (IL1, amino acids 97–102; IL2, amino acids 166–187; IL3, amino acids 259–282; and C-tail residues 361–420) were separately ligated into pAS2-1 and assayed for interaction with candidate MORIP cDNA clones in pACT2. Bait and prey plasmids were simultaneously co-transformed into *S. cerevisiae* strain MaV103 and interactions assayed for β-gal activity as described above.

### Glutathione S-transferase Pulldown

Glutathione S-transferase (GST) fusion proteins were constructed by separately ligating cDNAs encoding human AUP1 (residues 1–410), DOK4 (residues 1–326), CSN5 (residues 1–335), SIAH1 truncations (residues 91–282, 91–157, and 151–282 for mapping studies) or MOR-IL2 (residues 166–187) into the expression vector pGEX-4T-1 (Amersham Biosciences, Piscataway, NJ). cDNAs encoding the MOR-IL2 (residues 166–187), SIAH1 (residues 1–282) and RanBP9 (residues 170–381), were subcloned into the pET30a expression vector (Novagen, Madison, WI) containing an S-tag. Fusion proteins were induced in *Escherichia coli* strain BL21 (DE3) using the ZYP-5052 auto-induction media as described previously [28], [29]. GST fusion proteins bound to glutathione sepharose beads (GE Healthcare, Piscataway, NJ) were used to pull down S-tagged proteins from bacterial lysates as previously described [19], [23]. GST bound beads or unbound beads were used as negative controls. Eluted proteins were separated by SDS-PAGE and transferred to a polyvinylidene fluoride (PVDF) filter for Western blot analysis. The filter was probed with a horseradish peroxidase (HRP)-conjugated anti-S-tag antibody (1∶5000 dilution, Novagen, Madison, WI), and immunoreactivity detected by enhanced chemiluminescence with an ECL Plus kit (GE Healthcare).

### Cell Culture and Treatments

Human embryonic kidney (HEK) 293 cells stably transfected with either FLAG-tagged mu-opioid receptor (HEK-MOR), delta opioid receptor (HEK-DOR), or kappa opioid receptor (HEK-KOR) were maintained in Dulbecco’s Modified Eagle’s Medium (DMEM) supplemented with 10% fetal bovine serum (FBS) and 400 mg/mL G418. HEK-MOR and HEK-DOR cell lines were generously provided by Dr. Mark van Zastrow, University of California San Francisco [30]. HEK-KOR cells were a gift from Dr. Lee-Yuan Liu-Chen, Temple University School of Medicine [31].

For studies investigating MOR ubiquitination and degradation, HEK-MOR cells were grown to ∼80% confluence and treated with either 200 µM chloroquine (lysosomal inhibitor; Sigma-Aldrich, St. Louis, MO), 30 µM MG132 (w/DMSO; proteosomal inhibitor; UBPBio, Aurora, CO), 10 µM DADLE (a MOR agonist; [D-Ala2, D-Leu5]-Enkephalin acetate salt; Sigma-Aldrich), or a combination of these drugs for the indicated times.

Treatment times were based on previous experiments by Hislop et al. [32].

### Transfection, Immunoprecipitation, Western blotting and Knockdowns

For co-immunoprecipitation (co-IP) experiments, HEK-MOR, DOR, or KOR cells (stably expressing FLAG-tagged MOR, DOR, or KOR, respectively) were separately transfected with constructs encoding full-length MORIPs subcloned in the pCMV-Tag3B expression vector (Stratagene, La Jolla, CA) containing a myc-tag. For functional studies of SIAH proteins, HEK-MOR cells were transfected with an empty pCMC-tag3B vector, full length human SIAH1 cDNA (pCMC-Tag3B vector), full length SIAH2 cDNA (pcDNA3.1-HA, generously provided by Elizabeth Floyd, Louisiana State University), truncated SIAH1 cDNA (residues 91–282 in pCMC-Tag3B vector), or truncated SIAH2 cDNA (residues 122–324, pCMC-Tag3B vector). Transfections were carried out using Effectene transfection reagent (Qiagen, Valencia, CA). Cells were cultured for 48 hours in DMEM containing 10% FBS and then lysed in lysis buffer (20 mM Tris-HCl pH7.5, 150 mM NaCl, 1 mM Na_2_EDTA, 1 mM EGTA, 2.5 mM sodium pyrophosphate, 1 mM β-glycerophosphate, 1 mM Na_3_VO_4_, 1% Triton X-100 and 1mg/mL leupeptin) supplemented with protease inhibitor cocktail (Pierce, Rockford, IL). MOR, DOR, and KOR were immunoprecipiated from 1 mg total cell lysates using a polyclonal rabbit anti-FLAG antibody (Sigma-Aldrich) coupled to Protein-G Dynabeads (Invitrogen). For ubiquitination studies, the amount of lysate loaded into each immunoprecipitation reaction was normalized to MOR immunoreactivity in 10 µg of total cell lysate to ensure equal amounts of immunoprecipitated MOR. Western blot analysis of immunoprecipitated complexes was performed by using a monoclonal mouse anti-myc antibody (1∶5000 dilution; Millipore, Billerica, MA), mouse anti-HA antibody (1∶2500; Covance, Emeryville, CA), or mouse anti-ubiquitin antibody (VU-1;1∶2500; LifeSensors, Malvern, PA). Lysates from SIAH-transfected cells were additionally probed with a rabbit anti-FLAG antibody (1∶10,000; Sigma) for the detection of MOR and a chicken anti-GAPDH antibody (1∶10,000; Millipore), which served as a loading control. HRP-conjugated secondary antibodies were purchased from Jackson ImmunoResearch (West Grove, PA) and used at a 1∶20,000 dilution.

Mouse brain co-IPs were performed using freshly prepared brain lysates from C57BL6 female mice (see Tissue Preparation and Sample Analysis). Briefly, Protein-G MAG sepharose beads were used to preclear immunocomplexes from total brain lysates. Protein-G MAG sepharose beads were coated with rabbit anti-MOR antibody (AB1580, Millipore) according to the manufacturer’s instructions, and incubated with whole brain lysates. Eluted complexes were analyzed via SDS-PAGE/Western blotting using either goat anti-SIAH1 (1∶5000, Abnova), chicken anti-GPR177 (1∶2,500, [19]), or mouse anti-VAPA (1∶5000, Abnova) antibodies.

### Animal Care and Drug Treatment

All mouse studies were performed in the research laboratories at the Department of Veterans Affairs Medical Center in Coatesville, PA, and were approved by the Institutional Animal Care and Use Committee at both DVAMC Coatesville and the University of Pennsylvania. C57BL/6J mice were bred in-house, maintained on a 14 hr/10 hr light/dark schedule and had food and water available ad libitum throughout the course of the experiment.

Female mice, 8–9 weeks of age, were implanted sub-cutaneously with a 25 mg morphine (n = 5) or placebo (n = 4) pellet. Morphine and placebo pellets were obtained from the NIDA Drug Supply Program. Mice were euthanized by cervical dislocation under carbon dioxide anesthesia four days (96 hr) after pellet implantation. Brains were harvested and dissected on ice under 5× magnification into 6 regions including prefrontal cortex, nucleus accumbens, dorsal striatum, midbrain, hippocampus, and cerebellum. The atlas of Paxinos and Watson [33] was used as a guide. Specimens were frozen on dry ice and stored at −80°C.

### Tissue Preparation and Protein Analysis

Frozen brain tissue obtained from drug- or sham-treated mice was utilized for protein expression analysis. Fresh whole-brain tissue from untreated mice was obtained for co-IP experiments. All tissue was suspended in lysis buffer (50 mM Tris-HCl, 1 mM EDTA, 150 mM NaCl, 1% NP40, 0.25% deoxycholate, 5 mM NaF, 2 mM Na_3_VO_4_) containing protease inhibitors (cOmplete MINI EDTA free, Roche), homogenized using a microcentrifuge pestle for 2 minutes, sonicated using a probe sonicator, then centrifuged at 13,000 RPM to remove cellular debris. For protein expression analysis, samples were analyzed by electrophoresis on 10% SDS-containing polyacrylamide gels, followed by Western blotting. In addition to antibodies utilized for co-IP, guinea pig anti-MOR (GP10106, Neuromics, Edina, MN) and rabbit anti-DOK4 (SAB1300112, Sigma-Aldrich) antibodies were also used to probe brain lysates. For quantitative analysis of MORIP expression in drug treatment studies, lysates from sham and morphine-treated mice (20 µg/brain region) were analyzed in quadruplicate. To rule out transfer artifacts, samples from each brain region were loaded in a different order on each of the four blots. Blots were scanned using a back-lit scanner and quantification was performed using IMAGEJ software [34]. Expression was normalized to total protein (as measured by Ponceau stain), averaged between replicates, and subjected to a two-tailed Students t-test.

## Results

### Identification of Novel MORIPs

To identify novel mu-opioid receptor interacting proteins (MORIPs), we used the second intracellular loop of the MOR as bait to screen a fetal human brain cDNA library. In control experiments, the MOR-IL2 construct used in these studies did not autoactivate β-galactosidase expression (data not shown). We screened 2×10^6^ colonies and isolated 30 positive (β-gal+) clones representing eight distinct human proteins (Table 1). Sequence analysis identified several clones encoding components involved in the ubiquitin pathway and in signal transduction. These included COP9 subunit 5 (CSN5), a protein proposed to regulate exosomal protein sorting in both a deubiquitinating activity-dependent and -independent manner [35], ancient ubiquitous protein 1 (AUP1), a protein that promotes ubiquitination of misfolded proteins [36], and seven in absentia homologs 1 and 2 (SIAH1 and SIAH2), known RING Finger E3 ubiquitin ligases [37]. Four putative scaffolding proteins, docking protein 4 (DOK4), docking protein 5 (DOK5), Zyxin, and Ran binding protein 9 (RanBP9) were also identified as candidate MORIPs (Table 1).

To identify additional MORIPs, we performed a modified MYTH screen using the full-length MOR as bait [24], [25], [27], [38], [39], [40], [41]. In the initial MYTH screening, 104 positive clones were obtained from the 6×10^6^ colonies screened. Sequence analysis identified GPR177 (WLS; [19], a putative multi-pass membrane-spanning protein that is the mammalian ortholog of *Drosphila* Wntless/Evi/Sprinter [42]. WLS plays an important role in Wnt protein secretion, a process that is inhibited by the opioid agonist morphine [19]. Additionally, vesicle-associated membrane protein-A (VAPA or VAP33), connexin 37 (Cx37), and voltage-gated potassium channel Kv5.1 were also identified in the MYTH screen (Table 1). These proteins play a role in endoplasmic reticulum to Golgi transport [43], [44], gap junction formation [45], and regulation of resting membrane potential [46], respectively.

**Figure 1:**
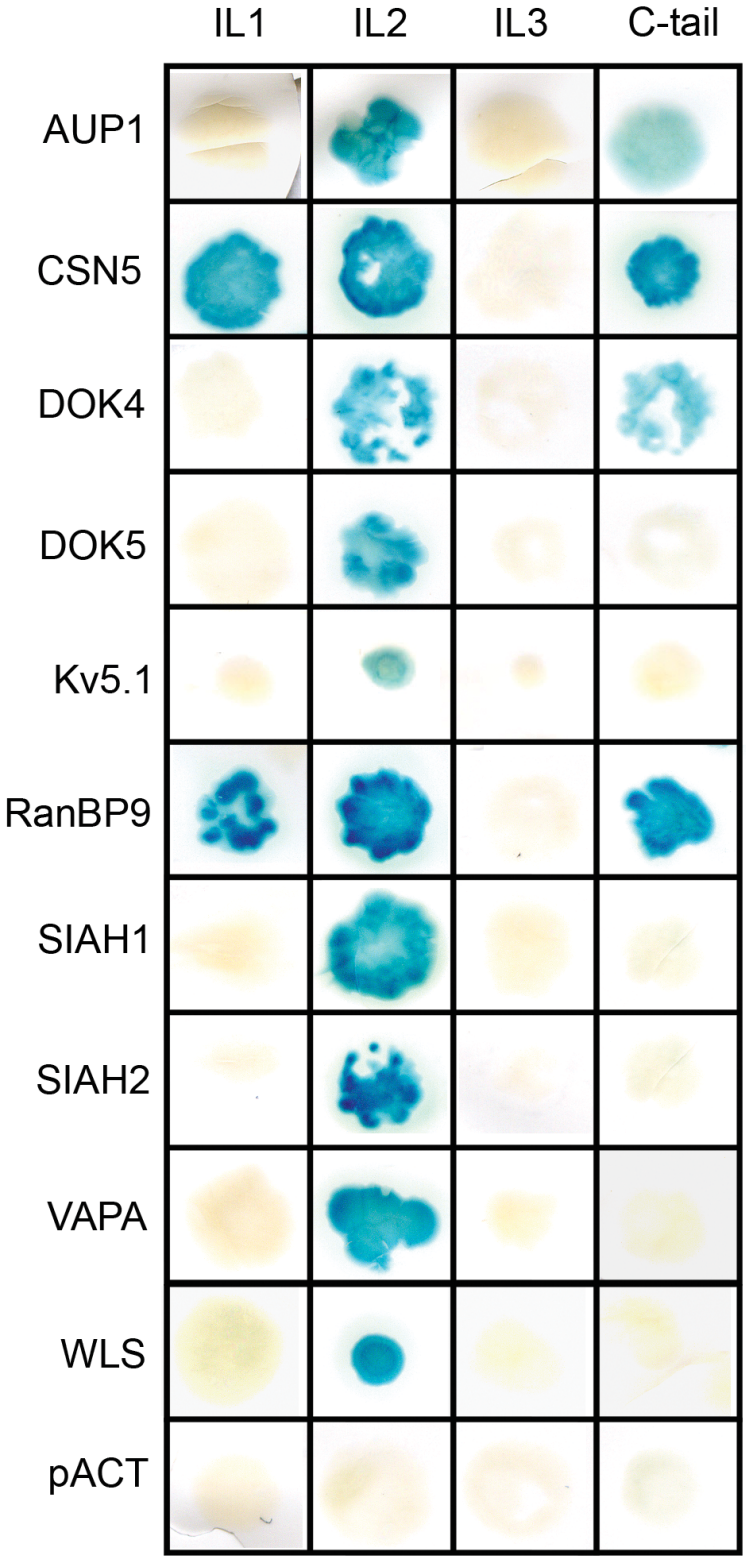
Mapping the MOR/MORIP interaction. Candidate MORIPs identified in the traditional and MYTH Y2H screens (preys) were tested for interaction with each of the intracellular loops (IL) and carboxyl-terminus (C-tail) of the mu-opioid receptor (baits) in a directed Y2H assay. An empty bait vector (pACT) was used as a negative control. A positive interaction is indicated by the production of a blue yeast colony in the β-galactosidase assay.

To map the binding site of the MORIPs on the MOR, we used the traditional yeast two-hybrid system to interrogate interaction of individual MORIPs with each of the MOR intracellular loops and the C-terminal cytoplasmic tail. Each of the MORIPs we identified interacted with IL2 of the MOR (Fig. 1 and Table 2). Two of the MORIPs, CSN5 and RanBP9, also interacted with MOR-IL1, while AUP1, CSN5, DOK4 and RanBP9 were found to interact with the C-tail of the receptor. The binding site for Cx37 was not tested in this assay. These results suggest that the MORIPs we identified interact with specific domains of the MOR in the yeast system, and that the second intracellular loop of the receptor is an apparent hot-spot for protein-protein interactions.

**Table 2:** Confirmation of MOR-MORIP interactions.

MORIP	IL1 Y2H^1^	IL2 Y2H^1^	IL3 Y2H^1^	C-tail Y2H^1^	GST PD^2^	DOR Co-IP^3^	KOR Co-IP^3^	MOR Co-IP^3^
AUP1	−	+	−	+	+	+	+	+
CSN5	+	+	−	+	+	−	−	+
Cx37	ND	ND	ND	ND	ND	+	+	+
DOK4	−	+	−	+	+	+	+	+
DOK5	−	+	−	−	ND	ND	ND	ND
Kv 5.1	−	+	−	−	ND	ND	ND	ND
RanBP9	+	+	−	+	+	ND	ND	ND
SIAH1	−	+	−	−	+	+	+	+
SIAH2	−	+	−	−	ND	+	+	+
VAPA	−	+	−	−	ND	+	+	+
WLS	−	+	−	−	+	+	+	+
Zyxin	ND	ND	ND	ND	+	ND	ND	ND

1 MORIPs isolated either in traditional or MYTH screens were tested for interaction with each of the intracellular loops (IL1, 2, or 3) or carboxyl-terminus (C-tail) of the MOR in a directed Y2H assay.

2 GST PD: Full-length MORIPs were tested for interaction with the MOR-IL2 in a GST pulldown assay.

3 Co-IP: Full-length MORIPs were tested for association with the delta (DOR), kappa (KOR), or mu (MOR) opioid receptor by co-immunoprecipitation.

+ indicates a positive result, - indicates a negative result, while ND indicates not done.

### Validation of Direct MOR/MORIP Interactions

**Figure 2:**
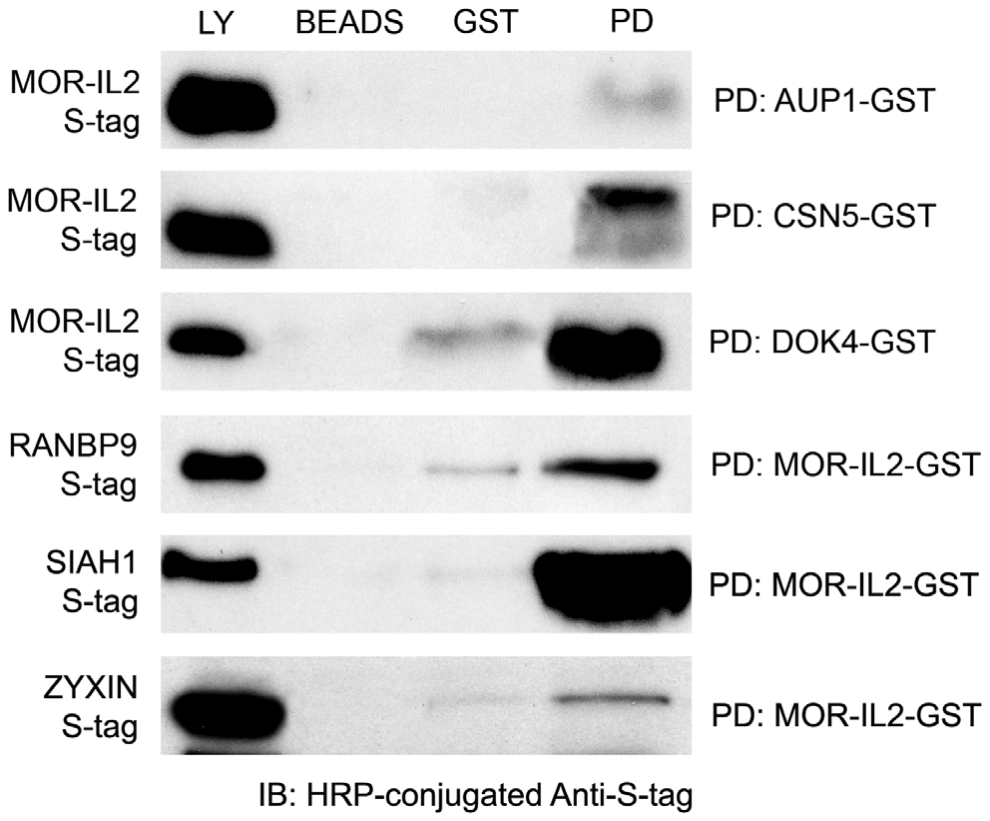
Confirmation of MORIP/MOR-IL2 interactions. GST pulldown assays were performed to interrogate the interaction between selected full-length MORIPs and the MOR-IL2 domain. In the top three panels, MORIP-GST fusion proteins were used to pull down the S-tagged MOR-IL2. In the bottom three panels, MOR-IL2-GST fusion proteins were used to pull down S-tagged MORIPs. Pulldown products were purified on glutathione beads, separated by SDS-PAGE, and probed on Western blots using HRP-conjugated anti-S-tag antibodies. S-tagged MORIPs or MOR-IL2 domains produced in bacteria are shown in lysate lanes (Ly), while uncoated glutathione sepharose beads (Beads) or GST-coated glutathione sepharose beads (GST) incubated with S-tagged proteins served as negative controls. PD indicates pull-down lanes.

To further validate direct interaction between newly identified MORIPs and the MOR, we tested the ability of selected MORIP-GST fusion proteins to associate with S-tagged MOR-IL2 in a pull-down assay. The results are shown in Fig. 2 and summarized in Table 2. Western blots containing lysates from bacteria expressing an S-tagged MOR-IL2 cDNA produced an immunoreactive band of ∼13 kDa when probed with anti-S-tag antibodies. This band corresponds to the expected size of the MOR-IL2 encoded by the cDNA construct. The same band was detected by pull-down after the bacterial lysate was incubated with the CSN5-GST, AUP1-GST, or DOK4-GST fusion proteins, but not when the lysate was absorbed onto beads alone or GST-coated beads. We also utilized the pull-down assay to test the ability of RanBP9, SIAH1, and Zyxin to associate with a MOR-IL2-GST fusion protein. As shown in Fig. 2, S-tagged RanBP9 cDNA produced an immunoreactive band of ∼34 kDa, S-tagged SIAH1 cDNA produced a band of ∼32 kDa, and S-tagged Zyxin was detected at ∼85 kDa when probed with anti-S-tag antibody. These bands correspond in size to the predicted sizes of the protein fragments encoded by the respective cDNA constructs, and were not detected when the lysates were absorbed onto beads alone or GST-coated beads.

### Interaction of MORIPs with MOR, DOR, and KOR in Cultured Mammalian Cells

**Figure 3:**
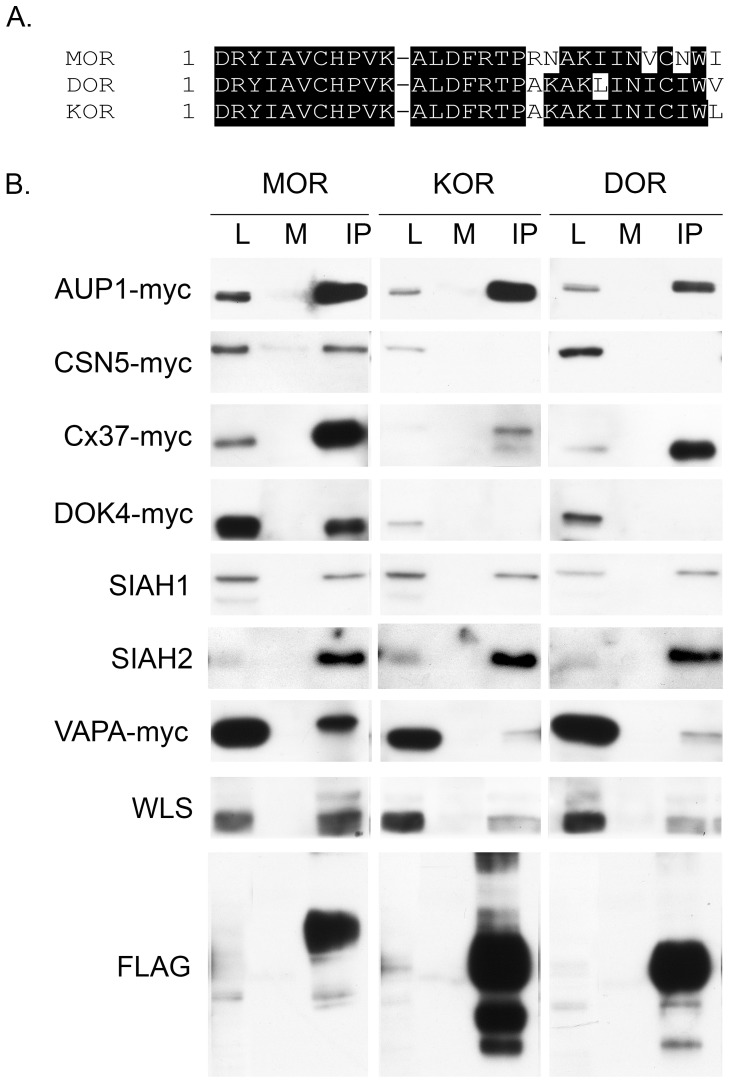
Interaction of MORIPs with MOR, DOR, and KOR in mammalian cells. (A) Amino acid sequence comparison between MOR-, DOR-, and KOR-IL2 domains. (B) Co-immunoprecipitation of full length MORIPs with MOR, DOR, or KOR. FLAG-tagged MOR, DOR, or KOR was immunoprecipitated from HEK-MOR, HEK-DOR, or HEK-KOR cells, respectively, using rabbit anti-FLAG antibodies. Mock immunoprecipitations were performed with Protein-G beads coated with non-specific rabbit IGG. Blots were probed with either anti-MORIP antibodies for the presence of endogenously expressed MORIPs (SIAH1, SIAH2 and WLS) or with anti-myc antibodies for transiently transfected myc-tagged MORIPs. A representative blot from each column was probed for mouse anti-FLAG to confirm immunoprecipitation of MOR, DOR, and KOR from these cell lines. Lysate lanes (L) contain 5% of the total protein compared to the mock (M) and immunoprecipitation (IP) lanes.

The interaction between full-length MOR and full-length MORIPs was verified using co-IP experiments. To demonstrate an interaction in cell culture, we tested the ability of an anti-FLAG antibody to co-IP MORIPs from lysates prepared from HEK-MOR cells that stably express FLAG-tagged MORs and have been transiently transfected with myc-tagged MORIPs. Probing Western blots with anti-myc antibodies revealed immunoreactive bands of the expected sizes in lysate lanes (L) prepared from HEK-MOR cells transiently transfected with myc-tagged AUP1, CSN5, Cx37, DOK4, SIAH1, SIAH2, VAPA, or WLS cDNAs (Fig. 3A). Bands migrating at similar molecular weights were detected in the co-IP lanes, but not in negative controls. Independent Y2H and co-IP experiments have recently confirmed an interaction between RanBP9 and the MOR [47]. Taken together, these results support the view that the MORIPs identified in our Y2H screens interact with full length MOR in the context of cultured mammalian cells (summarized in Table 2).

Our Y2H and pulldown data indicate that each of the MORIPs identified in our screens interacts with the MOR-IL2 (Fig. 1). Sequence comparisons indicate that the IL2 of the MOR, DOR and KOR exhibit a high degree of amino acid sequence similarity (Fig. 3A). Among the opioid receptor proteins, IL2 exhibits 85–90% amino acid sequence identity, with the greatest conservation within the N-terminal portion of the loop. Because of this homology, we used co-IPs to determine whether any of the MORIPs also interacted with other opioid receptor family members. To do this, we tested the ability of anti-FLAG antibodies to co-IP MORIPs from lysates prepared from HEK-DOR and HEK-KOR cells (stably expressing FLAG-tagged DORs and KORs, respectively) and separately transfected with each of the myc-tagged MORIPs. As shown in Fig. 3B and summarized in Table 2, AUP1, Cx37, SIAH1, SIAH2, VAPA, and WLS were able to interact with both DOR and KOR. However, neither CSN5 nor DOK4 showed interaction with DOR or KOR, despite the fact that the proteins were expressed in transfected HEK-DOR and HEK-KOR cells (Fig. 3B). Together, these results suggest that many of the MORIPs we identified are also capable of interaction with the DOR and KOR, at least within the context of transfected mammalian cells. Thus, it may be more precise to describe these interactors as ORIPs (opioid receptor interacting proteins), rather than MORIPs. The interaction of these proteins with each of the opioid receptor family members will depend in large part on whether an opioid receptor family member and a particular opioid receptor binding protein are co-expressed within the same cell (and cellular compartment) within the nervous system.

### Interaction of MOR and MORIPs in Rodent Brain

**Figure 4:**
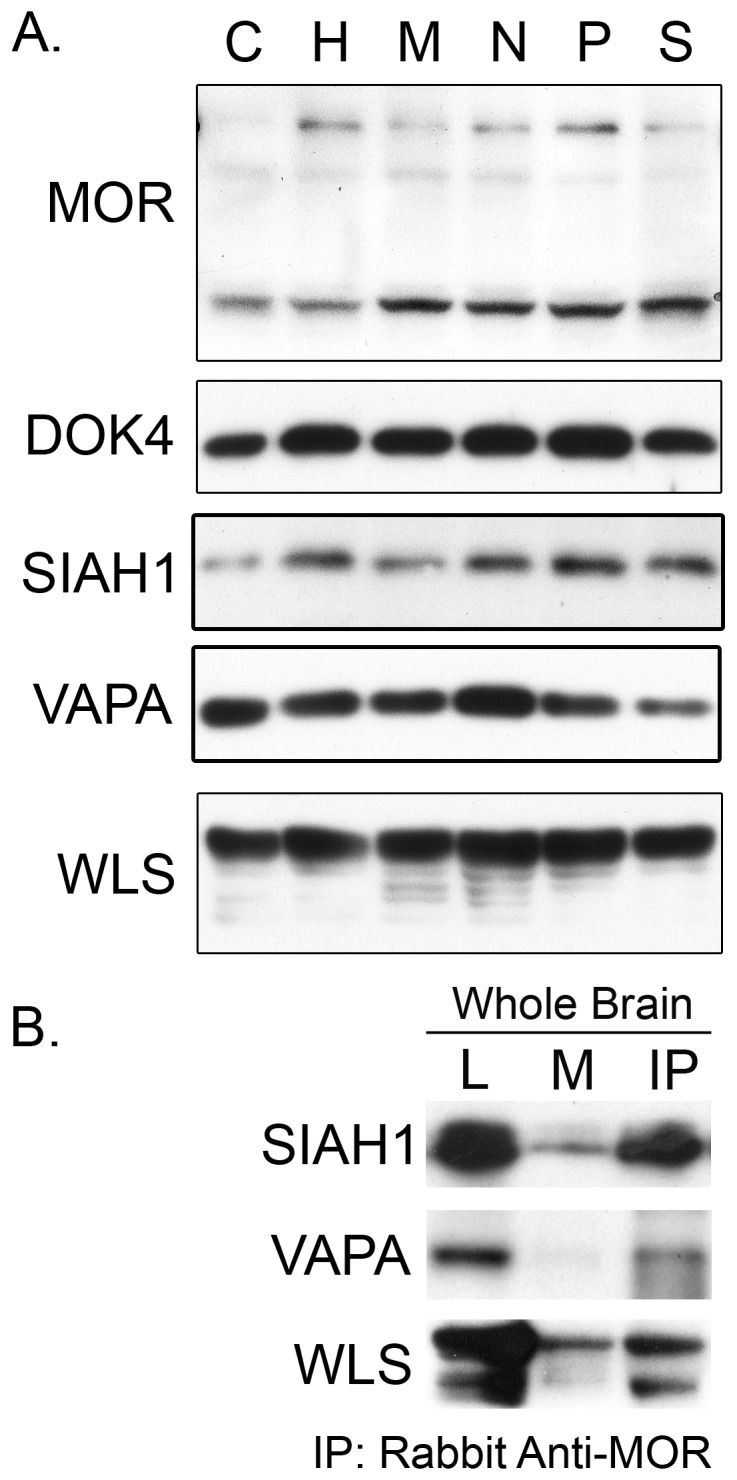
MORIP expression and interaction with MOR in mouse brain. (A) Expression of MOR and MORIPs in mouse brain regions. Western blots containing lysates prepared from mouse cerebellum (C), hippocampus (H), midbrain (M), nucleus accumbens (N), prefrontal cortex (P), and striatum (S) were probed with anti-MORIP antibodies. (B) Co-immuniprecipitation. The MOR was immunoprecipitated from whole mouse brain lysates using rabbit anti-MOR antibodies. Immunocomplexes were probed for the presence of SIAH1, VAPA, or WLS using MORIP-specific antibodies. Lysate lanes (L) contain 5% of the total protein prepared from whole mouse brain lysate compared to the mock (M, rabbit IGG) and immunoprecipitation (IP) lanes.

To determine whether the interaction between the MOR and MORIPs may occur *in vivo*, we analyzed the expression of MOR and several MORIPs in various mouse brain regions. As shown in Fig. 4A, the MOR and its interacting partners DOK4, SIAH1, VAPA, and WLS were each expressed in cerebellum, hippocampus, nucleus accumbens, midbrain, prefrontal cortex, and dorsal striatum. Co-expression of the MOR and each of the MORIPs in all brain regions tested is consistent with the idea that the MOR and its binding partners have the potential to interact in multiple brain regions. To verify interaction, the MOR was immunoprecipitated from a whole mouse brain lysate, and immunocomplexes probed for the presence of several MORIPs. As shown in Fig. 4B, SIAH1, VAPA, and WLS were able to co-IP with MOR but not with rabbit-IgG alone, suggesting that these MORIPs are capable of interacting with MOR in mouse brain. DOK4 was not tested due to crossreactivity of this antibody with rabbit IgG, while several other proteins were difficult to analyze due to the poor quality of available antibodies.

### The Effect of Morphine on MORIP Expression

**Figure 5:**
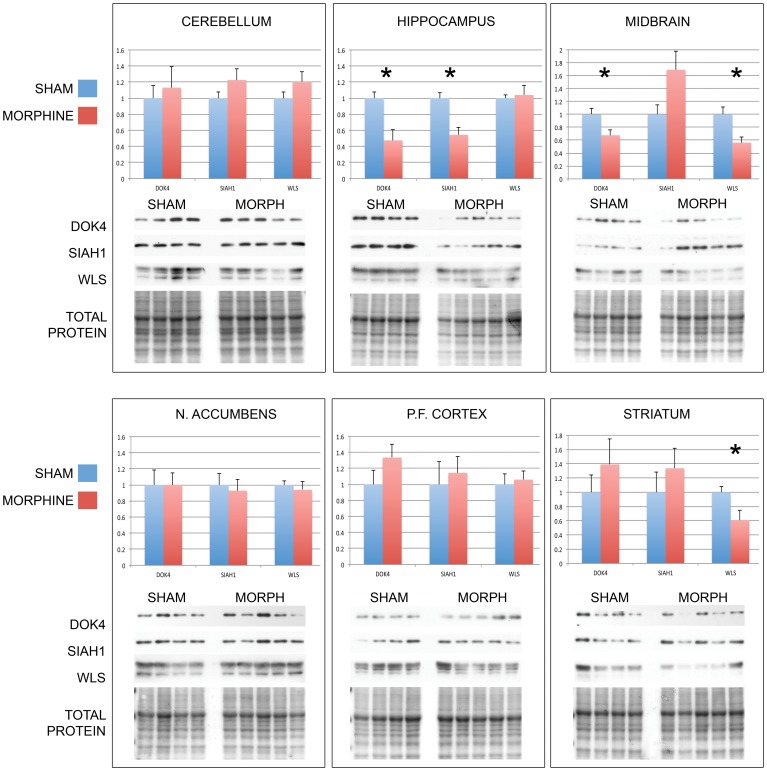
MORIP expression in brain regions of morphine-treated mice. Mice were treated for 96 hours with either morphine-containing (n = 5) or placebo (n = 4) pellets. Animals were sacrificed, brain regions dissected, and Western blots of selected MORIPs probed with MORIP-specific antibodies. Each panel contains a representative blot for a MORIP in the specified brain region (n = 4 blots/MORIP/brain region). Total protein was quantified by Ponceau stain of the blot prior to antibody probing. Bar graphs represent the average pixel density (as determined by imageJ) of four blots for each brain region normalized to total protein and placebo treatment. Data was analyzed using a two-sided Student’s t-test. Error is expressed as standard error of the mean. * indicates a statistically significant difference (p<.05) between sham and morphine treatment.

To determine whether chronic morphine exposure alters the protein levels of MORIPs, we performed Western blot analysis of selected MORIPs (DOK4, SIAH1, and WLS) in brain regions thought to be involved in response to opioid drug exposure. Mice were implanted subcutaneously with either saline (sham n = 4) or morphine (n = 5) pellets for 96 hours. As shown in Fig. 5, no significant changes were detected in any of the MORIPs tested in cerebellum, nucleus accumbens, or prefrontal cortex. However, in response to morphine exposure, DOK4 levels were significantly decreased by 53% in hippocampus (p = 0.01) and 33% in midbrain (p = 0.02). In morphine-treated animals, SIAH1 expression was reduced by 46% in hippocampus (p = 0.005). WLS levels were significantly decreased by 44% in midbrain (p = 0.01) and 30% in striatum (p = 0.03) following drug exposure. Together these data suggest that morphine treatment may lead to significant alterations in MORIP protein expression in various regions of mouse brain.

### SIAH 1 and SIAH2 do not Affect MOR Ubiquitination

MOR is known to be ubiquitinated on the first intracellular loop, which affects sub-endosomal localization of the receptor [32], [48]. SIAH1 and SIAH2, two MORIPs identified in the current study, are RING finger E3 ubiquitin ligases that have been shown to ubiquitinate a variety of cellular proteins, including several surface receptors, and target them for degradation [49], [50], [51], [52], [53], [54], [55], [56]. We, therefore, investigated whether SIAH1 and SIAH2 contribute to ubiquitination or degradation of the mu-opioid receptor.

While one previous study has suggested a role of the proteasome in agonist-induced down-regulation and basal turnover of mu and delta opioid receptors [57], it is generally believed that, like other GPCRs, MOR degradation is mediated primarily via the lysosomal degradation pathway [32], [58]. To confirm that this is the preferred mechanism of MOR proteolysis in the HEK-MOR cells used in our studies, we analyzed the effects of chloroquine (a lysosomal blocker) and MG132 (a proteosomal inhibitor) on steady-state MOR expression levels and in HEK-MOR cells stimulated with DADLE.

**Figure 6:**
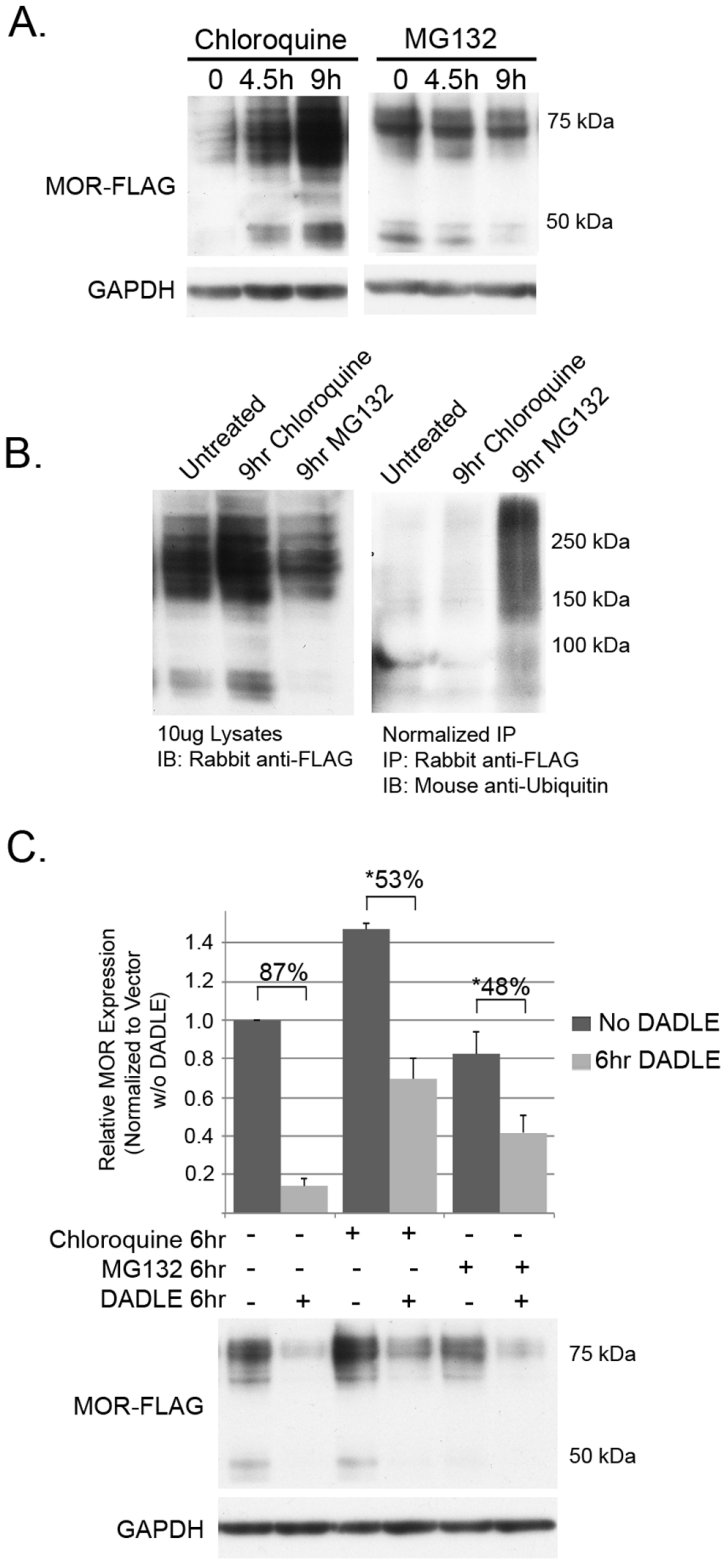
Regulation of MOR protein abundance and ubiquitination. (A) Lysates (10 µg) from HEK-MOR cells treated with 200 µM chloroquine or 30 µM MG132 for 0, 4.5, or 9 hours were probed with anti-FLAG antibodies. All blots were normalized to GAPDH. (B) Lysates from HEK-MOR cells treated for 9 hours with the indicated inhibitors were immunoprecipitated with rabbit anti-FLAG and blots probed for mouse anti-ubiquitin (right panel). The amount of lysate loaded into each IP reaction was normalized to the amount of MOR detected in 10 µg of cell lysate (left panel) to ensure that equal amounts of MOR were being immunoprecipitated. Experiments for A and B were performed in triplicate. (C) HEK-MOR cells were treated for 6 hours with no proteolytic inhibitors, chloroquine, or MG132 in the presence or absence of 10 µM DADLE. Total cell lysates (10 µg) were probed for rabbit anti-FLAG. Bar graphs represent the average pixel density (as determined by ImageJ) from 5 experiments normalized to GAPDH and an untreated control (no inhibitor or DADLE). The average percent DADLE-induced decrease in MOR levels for each inhibitor treatment was compared to the percent reduction observed without inhibitor treatment using a two-sided paired Student’s t-test. Error is expressed as standard error of the mean. * (p<.01) and ** (p<.005) indicate statistical difference as compared to no inhibitor treatment. IP indicates the antibody used for immunoprecipitation while IB indicates the antibody used for immunoblotting.

As shown in Fig. 6A, blocking the lysosomal degradation pathway with 200 µM chloroquine for 4.5 or 9 hours led to an increase in MOR protein levels, whereas blocking the proteasomal degradation pathway with 30 µM MG132 for similar times led to a reduction in MOR levels. These results are consistent with previous reports that MOR proteolysis occurs predominantly via the lysosomal degradation pathway [32], [58]. Ubiquitination of MOR species was analyzed by immunoprecipitating equal amounts (normalized to MOR levels in lysates) of MOR from cell lysates and probing for ubiquitin. It should be noted that with this protocol, it is possible that the ubiquitinated proteins detected may also include MORIPS that co-immunoprecipitate with MOR. Inhibition of lysosomal degradation with 200 µM chloroquine for 9 hours did not lead to an increase in ubiquitinated MOR (Fig. 6B). These results indicate that ubiquitination may not be required for lysosomal degradation of MOR. In contrast, we observed an increase in MOR ubiquitination after 9 hours of proteosomal inhibition with 30 µM MG132 (Fig. 6B). Because the proteasome is not required for basal turnover of MOR, we hypothesize that this increase may represent an indirect effect of stabilizing other proteins involved in the ubiquitination of MOR or an increase in the ubiquitination of associated MORIPS.

DADLE, a mu and delta opioid receptor agonist that internalizes and promotes ubiquitination of MOR [32], was used to determine whether ligand-induced receptor degradation in HEK-MOR cells is regulated via the proteosomal or lysosomal pathway. In HEK-MOR cells, 6 hours of DADLE treatment lead to a mean decrease in MOR protein levels of 87% (Fig. 6C). In chloroloquine- or MG132-treated cells, DADLE induced a 53% or 48% reduction in MOR protein levels, respectively compared to cells that were not treated with inhibitor. In contrast to previous studies that implicate either the proteosome or the lysosome in the degradation of MOR [32], [57], our results indicate that in HEK-MOR cells both pathways may contribute to agonist-induced MOR degradation.

**Figure 7:**
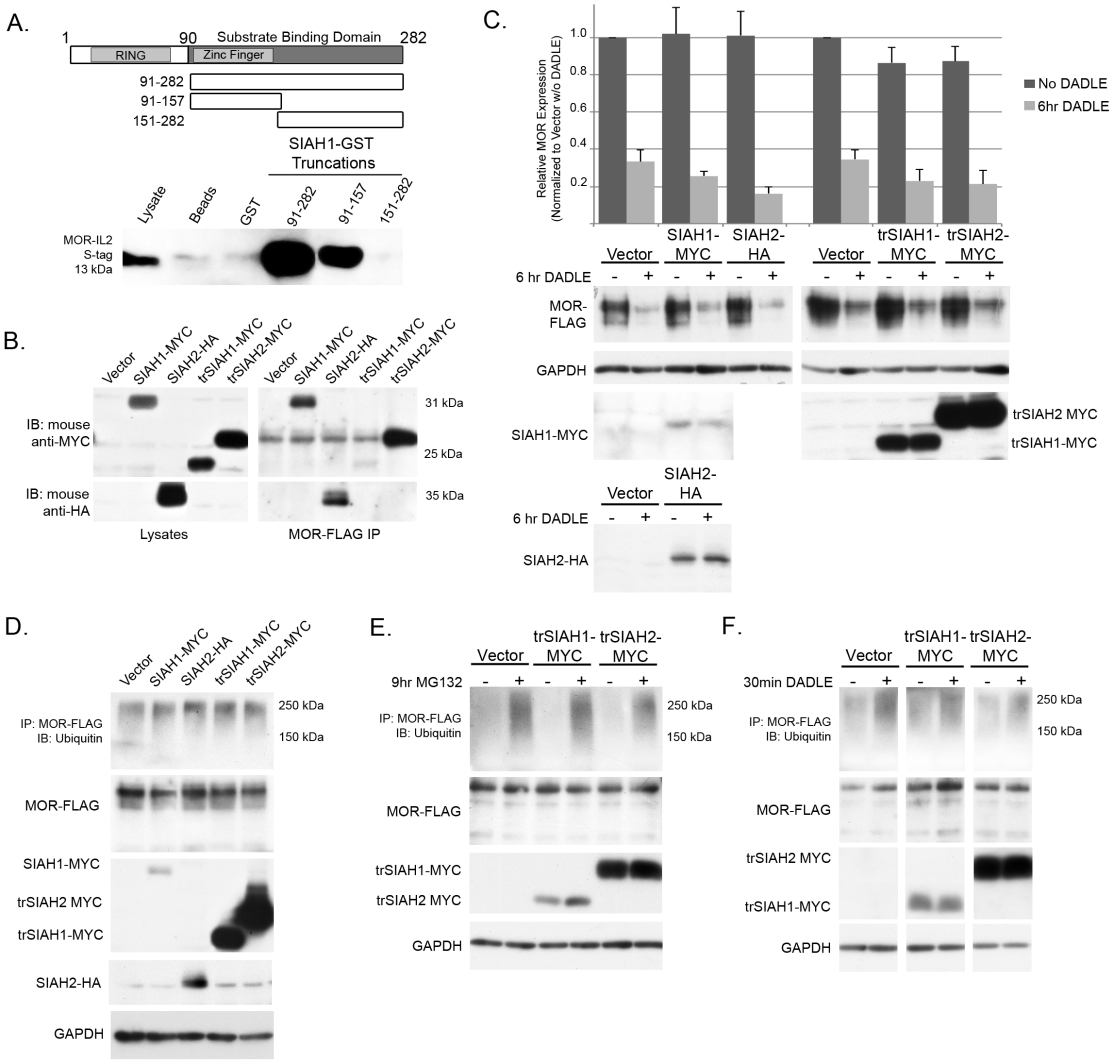
Role of SIAH1 and SIAH2 in regulating MOR ubiquitination. (A) Mapping the interaction site of MOR-IL2 on SIAH1 using GST-pulldowns. As controls, S-tagged constructs were incubated with untreated or GST-coated beads. (B) HEK-MOR cells were transfected with wild-type or truncated SIAH (trSIAH1 or trSIAH2) constructs and treated for 6 hours with 30 µM MG132. Proteins were immunoprecipitated and blots probed with either mouse anti-myc or mouse anti-HA to test for SIAH expression (left panels) and interaction with MOR (right panels), respectively. (C) HEK-MOR cells were transfected with wild-type or truncated SIAH constructs and either left untreated or treated with 10 µM DADLE for 6 hours. Blots were cut into sections and probed with rabbit anti-FLAG, mouse anti-myc, mouse anti-HA, or chicken anti-GAPDH antibodies. Bar graphs represent the average pixel density from 4 experiments normalized to GAPDH and untreated controls and subjected to a two-sided paired Student’s t-test. None of the SIAH constructs caused significant changes (at p<.05) in steady-state levels of MOR protein expression or in DADLE-mediated decreases in MOR expression levels. (D–F) Ubiquitination of MOR. Equal amounts of MOR (normalized from lysate blot) were loaded into immunoprecipitation reactions with anti-FLAG antibody. In each panel, the upper blot shows the IP probed for ubiquitin. All other blots show expression of various constructs or GAPDH in transfected cells. All experiments were performed in triplicate. (D) Steady-state ubiquitination levels in transfected HEK-MOR cells (E) Ubiquitination levels in transfected cells treated with 30 µM MG132 (F) Agonist induced ubiquitination in transfected cells treated with 10 µM DADLE.

Because proteasome blockers increase the ubiquitination state of MOR and can partially rescue DADLE-induced degradation of the receptor, we asked whether these processes involve the function of either SIAH1 or SIAH2. SIAH1 and SIAH2 are E3 ubiquitin ligases that contain an N-terminal catalytic RING finger domain and a C-terminal substrate-binding domain. GST pulldown assays with the second intracellular loop (IL2) of MOR show that the MOR-IL2/SIAH1 interaction is limited to the N-terminal half of the substrate-binding domain of SIAH1 (amino acids 91–157; Fig. 7A). Based on this mapping study, we generated SIAH1 and SIAH2 mammalian expression constructs lacking their respective RING domains. These constructs should maintain an interaction with MOR while losing ubiquitin ligase capabilities. As shown in Fig. 7B, wild-type and truncated versions of SIAH1 and SIAH2 were able to interact with the MOR as evidenced by their ability to co-immunoprecipitate with the receptor. It is known that wild-type SIAH proteins autoubiquitinate [59] leading to low levels of detectable protein. Therefore, the cells used for this experiment were treated for 6 hours with MG132 to prevent degradation of the transfected SIAH1 and SIAH2 proteins.

We next analyzed the effect of expressing wild-type or truncated SIAH1 and SIAH2 proteins on MOR levels in untreated and DADLE-stimulated HEK-MOR cells. Overexpression of wild-type or truncated SIAH proteins did not significantly alter steady-state MOR expression levels or DADLE-induced degradation of MOR (Fig. 7C). These results suggest that neither SIAH1 nor SIAH2 are involved in regulating MOR protein levels.

Data from this study and that of Hislop et al. [32] indicate that the ubiquitination state of the MOR is independent of protein levels and is not required or sufficient for receptor degradation. However, it is possible that SIAH proteins may serve to ubiquitinate the MOR as a requisite for their proper intracellular trafficking. To assess whether SIAH proteins may play a role in MOR ubiquitination, we first analyzed the effect of wild-type or truncated SIAH constructs on steady-state MOR ubiquitination. Results from this analysis indicate that neither the wild-type nor the truncated forms of SIAH1 or SIAH2 had any noticeable effect on the steady-state ubiquitination of MOR (Fig. 7D). Treatment of cells with 10 µM DADLE for 30 minutes [32] or 30 µM MG132 for 9 hours (Fig. 6C) leads to an elevation in MOR (and/or MORIP) ubiquitination. To determine whether SIAH1 or SIAH2 are responsible for this increase in ubiquitination, we assessed the effects of overexpressing truncated SIAH constructs on MOR ubiquitination in DADLE or MG132-treated HEK-MOR cells. As shown in Figs. 7E and 7F, expression of truncated SIAH1 or truncated SIAH2 did not block ubiquitination induced by MG132 or DADLE. Taken together, our results suggest that neither SIAH1 nor SIAH2 plays a direct role either in steady-state or agonist-induced degradation or ubiquitination of the MOR.

## Discussion

Using a combination of traditional Y2H and modified MYTH screening methods, we identified a novel cohort of mu-opioid receptor interacting proteins. In contrast to previous Y2H-based studies which employed either the IL3 or the C-tail of the MOR to discover potential binding partners, we used the MOR-IL2 as bait to screen a brain cDNA library. With this approach, we identified eight MORIPs. One of these interactors. RanBP9, has recently been confirmed as a MOR binding protein by an independent group [47]. Employing the entire MOR as bait in a MYTH screen, we identified four additional MORIPs, one of which (WLS) we have previously characterized in some detail [19], [20], [60], [61]. In contrast to the MORIPs identified in the traditional Y2H screen, each of the MORIPs isolated in the MYTH screen represents an integral membrane protein.

Because MYTH screens utilize the entire coding region of membrane associated proteins as bait, this method provides no information regarding the physical location of MORIP binding sites on the receptor. We therefore used the directed Y2H assay to first validate the interaction of the newly identified MORIPs with MOR, and then map the location and determine the specificity of the binding sites of these MORIPs on the receptor itself. Our studies showed that each of the MORIPs from the MYTH screen interacted specifically with the MOR-IL2 domain while several proteins from the traditional screen also interacted with additional intracellular portions of the receptor. Together, these mapping studies suggest that the MOR-IL2 represents a “hot spot” for protein/protein interactions. This is in accordance with our previous interaction studies using the D2 dopamine receptor (D2R), which showed that D2R- IL2 serves as a binding site for multiple interacting proteins [17], [62].

Sequence comparisons indicate that the IL2 domains of the MOR, KOR, and DOR are very highly conserved. Among the MORIPs tested, AUP1, Cx37, SIAH1, SIAH2, VAPA, and WLS also interacted with DOR and KOR in co-IP studies, suggesting that these MORIPs are likely to interact with the corresponding DOR and KOR-IL2 domains. As these protein interactions are not specific to any one subtype of opioid receptor it is likely that these proteins may be universal regulators of opioid function. Neither CSN5 nor DOK4 were found to interact with DOR or KOR. The failure of CSN5 and DOK4 to bind to DOR or KOR suggests that the non-conserved amino acids in the IL2 domains of these two opioid receptors may represent key residues that are necessary for the binding of CSN5 and DOK4 to the MOR. It is possible that these two MORIPs specifically regulate the function of MOR without affecting the function of DOR or KOR. The methods used to detect MOR/MORIP interaction in this paper were performed in the absence of agonist stimulation, which indicates that the interactions are ligand-independent. It is possible, however, that ligand stimulation would alter the MOR/MORIP interaction. This is the case with MOR and WLS where activation of the MOR with DAMGO or morphine enhances the MOR/WLS interaction [19].

**Figure 8:**
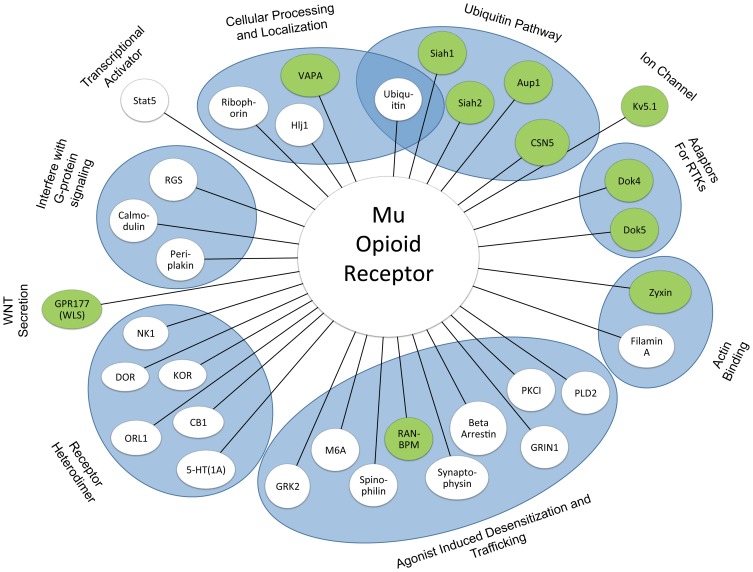
Functional categories of known and novel MORIPS. Newly identified and known MOR interacting proteins based on data from this and previous studies [18], [19], [46], [51], [66], [67], [68], [69], [70]. MORIPs were grouped based on established functional properties. MORIPs identified in the current study are depicted by green shaded circles, while previously identified MORIPs are represented by white circles. Many of the newly identified MORIPs also interact with DOR and KOR and may be considered general ORIPS (see Results).

The studies reported here add significantly to the list of known MOR binding partners, and as shown in Fig. 8, include proteins involved in virtually all aspects of MOR function including receptor-mediated signaling, trafficking, desensitization, cytoskeletal interaction, subcellular localization, degradation, and recycling. Of the novel proteins identified in our screens, we focused our attention on those of the DOK family of adapter proteins and those proteins involved in the ubiquitin/proteasome pathway.

Two proteins from the downstream of tyrosine kinase (DOK) family, DOK4 and DOK5, were identified as MORIPs using a traditional Y2H screening approach. This family of proteins has a broad range of functions. In the nervous system, DOK4 has been shown to be required for axon myelination [63] and the regulation of GDNF-dependent neurite outgrowth (through activation of the Rap1-ERK1/2 pathway) [64]. DOK5 interacts with TrkB and TrkC neurotrophin receptors, and is involved in the activation of the MAPK pathway induced by neurotrophin stimulation [65]. Neurotrophins, like opioid receptors, have been linked to pain reception, reward, and synaptic plasticity [66], [67]. For example, BDNF (a ligand of TrkB) has been implicated in substance dependence as well as antinociception [68], [69], [70], [71]. It will clearly be of interest to determine whether there is a functional interaction between the MOR and neurotrophic factors in MAPK-mediated signaling, pain processing, reward, and/or memory formation.

Several of the novel MORIPs identified in the current Y2H screens are components of the ubiquitin/proteasome pathway. Seven in absentia homologs 1 and 2 (SIAH 1 and 2) are RING finger E3 ubiquitin ligases that mediate ubiquitination and subsequent proteasomal degradation of target proteins [37]. CSN5 (COP9 subunit 5 or COP9S5) is a subunit of the large COP9 signalosome (CSN) which is responsible for the deneddylation and activation of Cullin RING E3 ubiquitin ligases that ubiquitinate target proteins for proteolysis [72]. Interaction of the MOR with these components of the ubiquitin/proteosome pathway was somewhat unexpected given the fact that the MOR is typically thought to be downregulated via lysosomal degradation [32]. However, recent studies have shown that upon internalization, the MOR is ubiquitinated in the first intracellular loop, and that this ubiquitination is involved in clathrin coated pit scission, endocytic sorting of the receptor, and down-regulation of receptor ligand binding [32], [73]. Since SIAH1 and SIAH2 are known E3 ubiquitin ligases [74], and each is capable of interacting with the MOR, we asked whether either protein contributed to the ubiquitination or degradation of the MOR. Although our results suggest that both the lysosomal and the proteosomal pathways contribute to agonist-induced decreases in MOR expression levels, SIAH1 and SIAH2 do not appear play a role in this process. Overexpression of SIAH1, SIAH2, or truncated dominant negative forms of either protein, did not affect MOR protein or ubiquitination levels in unstimulated cells, agonist stimulated cells, or cells treated with lysosomal or proteasomal inhibitors. Recently, it has been shown that the E3 ligase, Smurf2 is involved in ligand-induced ubiquitination of MOR [73]. Taken together, these studies argue against a role for the SIAH proteins in the regulation of MOR ubiquitination. Alternatively, it is possible that the MOR itself may serve as a scaffold to bring other MORIPs within the MOR signalplex into contact with the ubiquitination machinery. A potential example of this is the vesicle-associated protein synaptophysin, a previously identified MORIP that is a known ubquitination target of both SIAH1 and SIAH2 [51].

Knockout mouse experiments have shown that MOR is the primary mediator of the analgesic and rewarding properties of opioid drugs [1]. Since MORIPs regulate the functional output of the MOR, they represent potential candidates for proteins that may contribute to the underlying molecular mechanisms leading to drug addiction. Protein expression analysis of selected brain regions from morphine-treated mice indicate that the expression of DOK4, SIAH1, and WLS are altered in distinct brain regions following chronic morphine exposure. It is not known at this time whether these changes in expression are due to changes in protein stability, transcription, or translation.

The MOR/WLS interaction, which was originally identified in the same MYTH screen reported here, has previously been functionally characterized in HEK-MOR cells by our laboratory [19], [20]. We showed that morphine treatment promotes an increase in the MOR/WLS interaction at the plasma membrane. This in turn may be responsible for the decrease in Wnt protein secretion that occurs following morphine treatment. Thus it is tempting to speculate that the decrease in WLS expression observed in midbrain and striatum of mice after morphine administration could possibly lead to long term decreases in Wnt secretion within the CNS. Decreased Wnt secretion could therefore be responsible for many of the observed effects of chronic opioid exposure including decreased neurogenesis and decreased dendritic spine density that are known to be affected by Wnt signaling [75], [76]. Although the functional relevance of changes in WLS, DOK4 and SIAH1 expression after morphine exposure is currently unknown, it is of interest that these changes in protein expression were observed within specific brain regions, rather than throughout the entire brain. Thus the changes in protein expression we detected may play an important role in the response to opioid drug exposure mediated by these brain regions. In the future, it should become possible to examine the role of these MORIPs in behavioral aspects of opioid addiction using animal models of opioid self-administration.

The results presented in this study highlight the use of multiple screening methods to expand our knowledge of proteins that interact with, and contribute to the regulation of opioid receptor-mediated signaling in brain. Identification of the full panorama of MORIPs represents a critical step in understanding the normal regulation of the receptor life-cycle, as well as how receptor signaling or trafficking may be hijacked in response to drugs of abuse. The MORIPs identified in this and other studies may also represent key players involved in other opioid-mediated processes such as neural development, nociception, aversion, and synaptic plasticity. As the list of MORIPs grows, and their contributions to receptor function become better understood, it is possible that some of these proteins will also become targets for new drug development to prevent and/or treat opioid addiction.
